# Complementary vibrational spectroscopy

**DOI:** 10.1038/s41467-019-12442-9

**Published:** 2019-09-27

**Authors:** Kazuki Hashimoto, Venkata Ramaiah Badarla, Akira Kawai, Takuro Ideguchi

**Affiliations:** 1 Department of Physics, The University of Tokyo, Tokyo, 113-0033 Japan; 2 Aeronautical Technology Directorate, Japan Aerospace Exploration Agency, Tokyo, 181-0015 Japan; 3 Institute for Photon Science and Technology, The University of Tokyo, Tokyo, 113-0033 Japan; 4 PRESTO, Japan Science and Technology Agency, Saitama, 332-0012 Japan

**Keywords:** Infrared spectroscopy, Raman spectroscopy, Atomic and molecular interactions with photons

## Abstract

Vibrational spectroscopy, comprised of infrared absorption and Raman scattering spectroscopy, is widely used for label-free optical sensing and imaging in various scientific and industrial fields. The two molecular spectroscopy methods are sensitive to different types of vibrations and provide complementary vibrational spectra, but obtaining complete vibrational information with a single spectroscopic device is challenging due to the large wavelength discrepancy between the two methods. Here, we demonstrate simultaneous infrared absorption and Raman scattering spectroscopy that allows us to measure the complete broadband vibrational spectra in the molecular fingerprint region with a single instrument based on an ultrashort pulsed laser. The system is based on dual-modal Fourier-transform spectroscopy enabled by efficient use of nonlinear optical effects. Our proof-of-concept experiment demonstrates rapid, broadband and high spectral resolution measurements of complementary spectra of organic liquids for precise and accurate molecular analysis.

## Abstract (web summary)

Generally infrared and Raman spectroscopic methods are needed to study the symmetric and asymmetric molecular vibrational modes. Here the authors demonstrate complementary vibrational spectroscopy to organic molecules by simultaneously measuring their symmetric and anti-symmetric vibrations with one setup.

## Introduction

Vibrational spectroscopy is a fundamental method for chemical analysis used in a variety of scientific fields such as organic/inorganic chemistry, geology, biomedical, material, food, environmental, and forensic science^1–5^. The label-free noninvasive molecular spectroscopy enables us to acquire bond-specific chemical information of specimen, and it is known that infrared (IR) absorption and Raman scattering spectroscopy provide complementary information of molecular vibrations: the former is active for anti-symmetric vibrations that alter the dipole moment, while the latter for symmetric vibrations that alter the polarizability^1^. IR absorption spectroscopy, which is active for polar bonds such as O–H or N–H, is often used for identification of functional groups of molecules, while Raman scattering spectroscopy, active for bonds such as C=C, S–S, or C–S^4^, is used for identification of skeletal structures. The group theory states that fundamental vibrational modes of molecules with the center of symmetry cannot be both IR and Raman active (which is known as the rule of mutual exclusion^1,6^, while there are some exceptions^6,7^). Therefore, to acquire the complete information of molecular vibrations for more accurate and precise chemical analysis, both the IR and Raman spectra must be measured. Measuring the complete information of molecular vibrations enables us to analyze complex molecular phenomena such as catalytic chemical reactions^8–11^.

Simultaneous measurement of IR and Raman spectra is a grand challenge in spectroscopy because wavelength regions of these two spectroscopy methods are largely separated, that is, mid-infrared (2.5–25 µm, corresponding to 400–4000 cm^−1^) for IR spectroscopy and visible to near-infrared (0.4–1 µm, corresponding to 10,000–25,000 cm^−1^) for Raman spectroscopy, respectively. Since this large wavelength discrepancy causes the difficulty of sharing light sources and optics, a primitive combination of conventional Fourier-transform infrared spectroscopy (FT-IR) and Raman spectrometers^12,13^ has never been a convincing approach. Such a system requires a complex instrument comprises different spectroscopy methods based on a Michelson interferometer and a dispersive spectrometer with two independent light sources such as an incoherent lamp source and a visible continuous-wave laser. Additionally, these conventional methods do not provide state-of-the-art sensitivity and data acquisition speed because of the low brightness of the lamp source for FT-IR (especially when spatial mode filtering is required to have a small focusing spot) and the inherent weakness of spontaneous Raman scattering. Meanwhile, the technical advancement of nonlinear optics based on ultrashort pulsed lasers has enabled us to have higher brightness of coherent IR sources and stronger Raman signals through the coherent Raman scattering^5,14^, and some approaches have been made towards IR/Raman dual-modal spectral acquisition with a single pulsed laser^15,16^. However, these techniques neither have capability of simultaneous acquisition of complementary IR/Raman spectra nor broadband and high-resolution spectral acquisition covering the molecular fingerprint region (800–1800 cm^−1^), where the richest vibrational modes exist.

Here, we propose and demonstrate a simple yet powerful technique, called complementary vibrational spectroscopy (CVS), that allows us to simultaneously measure broadband IR and Raman spectra covering the fingerprint region at the same position. CVS is dual-modal Fourier-transform spectroscopy (FTS) enabled by an ultrashort near-infrared (NIR) pulsed laser and a Michelson interferometer. The IR spectroscopy is implemented as FT-IR with a coherent mid-infrared (MIR) pulsed source generated via intra-pulse difference-frequency generation (IDFG) from the NIR pulses^17–19^, while the Raman spectroscopy as Fourier-transform coherent anti-Stokes Raman scattering spectroscopy (FT-CARS) with the same NIR pulses^20^. The former uses a second-order and the latter a third-order nonlinear phenomena, respectively. The system is simple and robust because it shares a single laser source and an interferometer. Note that our proposed method can be applied to advanced FTS techniques such as dual-comb spectroscopy^21–23^, empowering the emerging technique further in respect to data acquisition rate, spectral resolution, and frequency accuracy.

## Results

### Principle of CVS

**Fig. 1:**
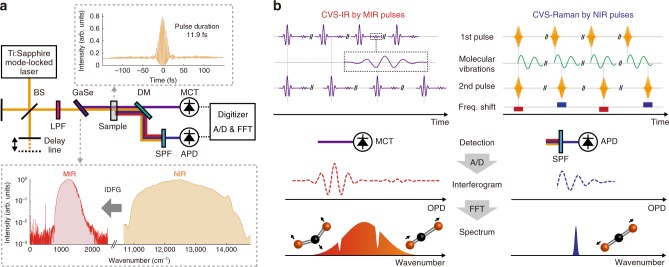
Schematic and concept of CVS. **a** Schematic of CVS. The insets show the autocorrelation trace of the NIR pulses and the spectra of NIR and MIR pulses. BS: Beamsplitter, LPF: Long-pass filter, DM: Dichroic mirror, SPF: Short-pass filter, MCT: HgCdTe, APD: Avalanche photodetector. **b** Conceptual description of CVS. The figure shows a linear triatomic molecule as an example of molecular vibrations. The left panel displays CVS-IR process given by MIR pulses, while the right panel CVS-Raman process by NIR pulses. OPD: Optical path length difference

The schematic representation of the system is shown in Fig. 1a. In CVS, both FT-IR and FT-CARS are implemented in a single FTS system that consists of a Michelson interferometer with a NIR femtosecond laser (10-fs Ti:Sapphire mode-locked laser at a repetition rate of 75 MHz in this study) as a light source. The pulses emitted from the laser are coupled in the interferometer and double NIR pulses are created from each pulse with an optical path length difference (OPD) set by the delay line in the interferometer. The NIR double pulses are focused onto a *χ*^(2)^ nonlinear crystal (GaSe crystal in this case) and a portion of the NIR pulses are converted to MIR pulses through the IDFG process. The generated MIR and undepleted NIR pulses collinearly irradiate the sample. The MIR pulses are absorbed, while the NIR pulses are inelastically (Raman) scattered by molecules. The MIR and NIR pulses are spatially separated by a dichroic mirror after passing through the sample and are simultaneously detected by a HgCdTe (MCT) photodetector and a Si avalanche photodetector (APD), respectively. Here, the NIR pulses are optically filtered before the detector so that only the blue-shifted scattered photons reach the APD. The detected signals are A/D-converted by a digitizer and the digitized interferograms are Fourier-transformed. The full schematic of the CVS is depicted in Supplementary Fig. 1.

The working principle of CVS is shown in Fig. 1b. In CVS-IR process, the MIR double pulses are modulated by IR-active molecular absorptions and their optical interference is detected by the MIR detector. Since the delay between the first and second MIR pulses is determined by that of the NIR pulses, the MIR absorption interferogram is measured as a function of the OPD between the NIR double pulses. Fourier-transforming the IR interferogram shows a broadband IR spectrum. On the other hand, in CVS-Raman process, the first NIR pulse excites the molecular vibrations and the second NIR pulse probes them and generates blue-shifted photons via anti-Stokes Raman scattering. By scanning the OPD between the NIR pulses, optical frequency of the second NIR pulse can be shifted by the refractive index modulations caused by the Raman active molecular vibrations induced by the impulsive stimulated Raman scattering process. A CARS interferogram that represents the molecular vibrations is obtained as an intensity modulation of the blue-shifted part of the second NIR pulses, which can be separated out by the optical short-pass filter. Finally, a broadband Raman spectrum is obtained by Fourier-transforming the CARS interferogram.

### Characterization of NIR and MIR pulses

We first characterize the NIR and MIR pulses. The spectrum of our 10-fs Ti:Sapphire laser spans over 10,870–14,490 cm^−1^ (690–920 nm) at the center wavelength of 12,500 cm^−1^ (800 nm), and its pulse duration is evaluated by autocorrelation measurement as 11.9 fs at the sample position. This ultrashort NIR pulses with the broadband spectrum spanning more than 3400 cm^−1^ allows us to measure broadband FT-CARS spectrum covering the molecular fingerprint region (800–1800 cm^−1^) and C–H stretching region (2800–3300 cm^−1^)^24^. The spectrum of the MIR pulses generated by the IDFG process in a 30-µm GaSe crystal is measured by a homemade FT-IR spectrometer and it spans from 790 to 1800 cm^−1^, which covers the fingerprint region. In this study, the lowest wavenumber of the IR spectrum is limited by the detection range of the MCT detector and the highest wavenumber is possibly limited by the phase-matching condition of the IDFG process. Note that the IR spectral region can be shifted by changing the angle of the crystal and also expanded by changing the crystal and/or laser system.

### Complementary vibrational spectroscopy

**Fig. 2:**
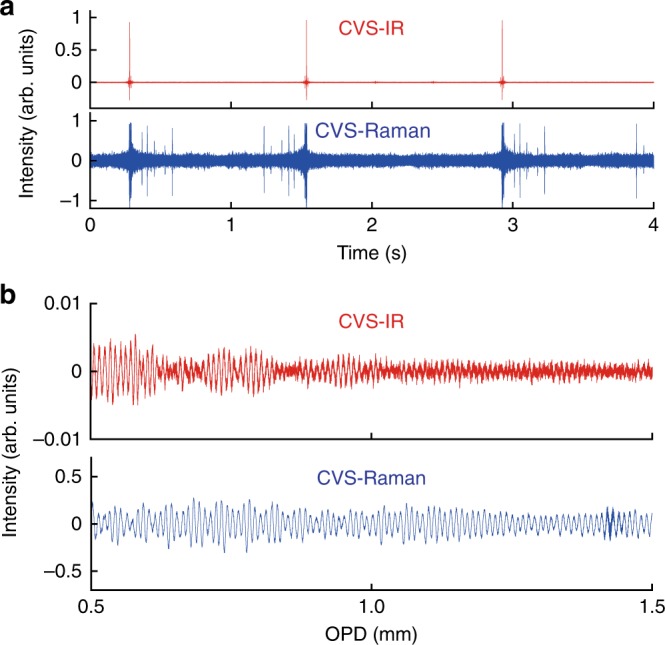
CVS interferograms of toluene. **a** The upper and lower panels show the sequential interferograms measured by CVS-IR and CVS-Raman spectroscopy, respectively. **b** Magnified 15-averaged CVS-IR and CVS-Raman interferograms plotted as a function of the OPD

As a proof of concept demonstration, we measure complementary vibrational spectra of liquid toluene. Figure 2a shows sequential CVS interferograms of toluene, where the IR and CARS interferograms are simultaneously detected. They show the synchronized bursts at zero-path-difference (ZPD) of the interferometer. The OPD is scanned over 2 cm at a rate of 0.8 Hz. Figure 2b shows 15-times coherently averaged IR/CARS interferograms, which clearly show signature of molecular vibrations. In the configuration where the MIR pulses are generated after the NIR interferometer, the raw IR temporal waveform contains components other than the desired IR interferogram. A detailed retrieval procedure of the IR interferogram is described in Supplementary Note 4 and Supplementary Fig. 3. The double-sided IR interferogram is apodized and Fourier-transformed, whereas the single-sided CARS interferogram is apodized and Fourier-transformed by omitting the center-burst caused by the non-resonant four-wave mixing process at ZPD.

**Fig. 3:**
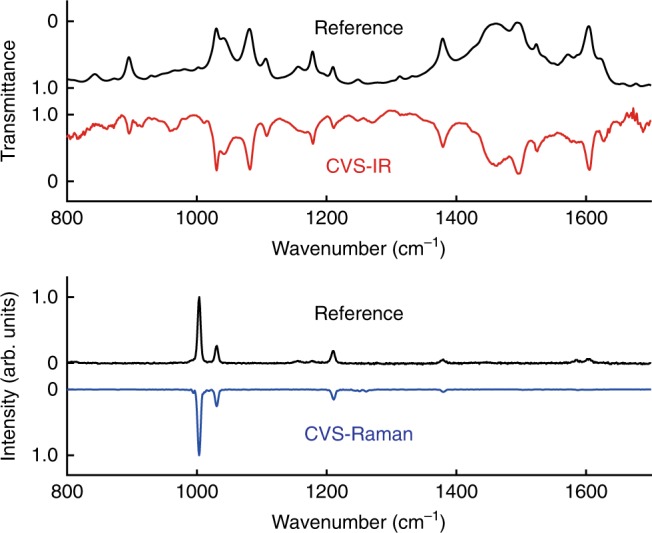
Complementary vibrational spectra of toluene in the fingerprint region. The upper panel shows the comparison of the CVS-IR spectrum and the reference IR absorption spectrum measured by a standard FT-IR. The lower panel shows the comparison of the CVS-Raman spectrum and the reference Raman scattering spectrum measured by a spontaneous Raman spectrometer

The complementary vibrational spectra Fourier-transformed from the interferograms shown in Fig. 2 are displayed in Fig. 3 with the reference spectra individually measured by conventional spectrometers. The upper panel shows the CVS-IR spectrum together with the reference spectrum measured by a standard FT-IR spectrometer (FT/IR-6800, JASCO). The CVS-IR transmittance spectrum of toluene agrees well with the reference spectrum and clearly displays the vibrational modes of, for example, C–H bending at 896, 1179, and 1495 cm^−1^, ring stretching at 1030, 1082, and 1605 cm^−1^, CH_3_ rocking at 1042 cm^−1^, C–CH_3_ stretching at 1211 cm^−1^ and CH_3_ deformation at 1379 and 1462 cm^−1^ (ref. ^25–28^) with a triangular-apodized spectral resolution of 3.5 cm^−1^. The lower panel shows the CVS-Raman spectrum and the reference spectrum measured by a standard spontaneous Raman spectrometer (inVia, Renishaw). The CVS-Raman spectrum at the apodized spectral resolution of 5.5 cm^−1^ clearly shows the vibrational modes of ring stretching at 1003 cm^−1^ and 1030 cm^−1^, C–CH_3_ stretching at 1211 cm^−1^ and CH_3_ deformation at 1380 cm^−1^^25,27,28^, which also agrees well with the reference spectrum. Note that the wavenumbers of the CVS spectra are calibrated with the same interferometer, so that we can compare the spectra in a precise manner. The sensitivity evaluation of the CVS is described in Supplementary Note 5 and Supplementary Figs. 4 and 5.

**Fig. 4:**
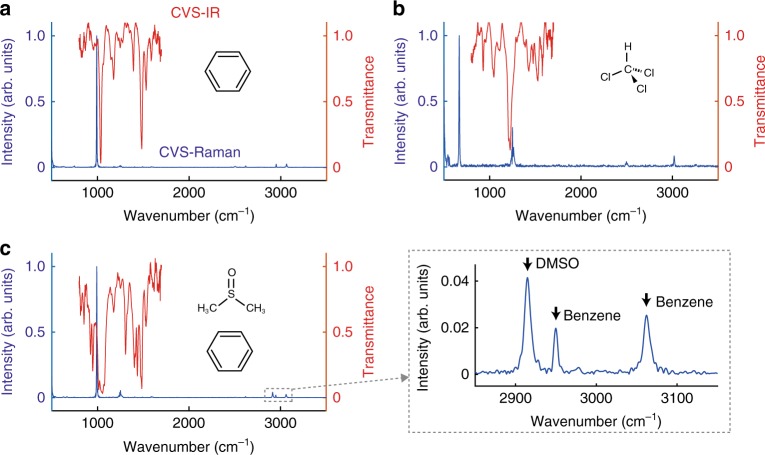
Complementary vibrational spectra of organic molecules. **a** benzene, **b** chloroform, **c** 4:1 mixture of benzene and DMSO. Red and blue curves represent CVS-IR and CVS-Raman spectra, respectively. The inset shows the zoomed spectrum of the Raman peaks of DMSO and benzene around 3000 cm^−1^. The vibrational lines are found from 800 to 1700 cm^−1^ in the CVS-IR spectrum and from 600 to 3100 cm^−1^ in the CVS-Raman spectrum. The small spikes at 500–550, 750, 1250, and 2500 cm^−1^ in the CVS-Raman spectra are attributed to instrumental noise, which can be removed by careful instrumentation

To show the applicability of this system for other samples, we measure the complementary spectra of three different kinds of liquid samples: benzene, chloroform and a 4:1 mixture of benzene and dimethyl sulfoxide (DMSO) (Fig. 4). Here, we show the CVS-Raman spectra up to around 3000 cm^−1^, showing its ultra-broadband measurement capability. This capability of measuring ultra-broadband spectra with high spectral resolution is a unique feature given by using Fourier-transform spectroscopy technique. Detailed assignment of these spectra are discussed in Supplementary Note 2. The small spikes at 500–550, 750, 1250, and 2500 cm^−1^ in the CVS-Raman spectra are attributed to the instrumental noise, which is discussed in Supplementary Note 2 and Supplementary Fig. 2.

## Discussion

Our CVS technique can be improved further. The measurable spectral span of the IR absorption spectrum (limited to 1000 cm^−1^ in this study) can be largely expanded by using other nonlinear crystals or light sources^29–32^, and/or by implementing the EO-sampling technique for detecting the IR interferograms^19,30,33^. In addition, since the CVS is based on FTS, it can be more rapid, robust and compact by using dual-comb FTS^21–23^ or ultra-rapid-scan FTS^24,34–36^. The higher scan rate allows us to measure dynamically changing complex phenomena or hyper-spectral multi-modal wide-area images. Furthermore, we could use the second harmonic pulses generated from the nonlinear crystal, together with the IDFG pulses for adding other spectroscopic modalities in CVS.

The CVS have the potential to provide means of chemical analysis. The simultaneous measurement of IR and Raman broadband spectra at the same spot can be essential for studying dynamic change in structure of complex molecules^11^. In addition, the highly accurate and common frequency axis for IR and Raman spectra would allow us to have fundamental study on molecules via precise analysis on relative line position or peak intensity between the IR and Raman spectra, which is essential for, for example, determination of molecular symmetry^37,38^ or two-dimensional correlation spectroscopy^39,40^.

## Methods

### NIR interferometer

A detailed schematic of the CVS is shown in Supplementary Fig. 1. The system is based on dual-modal FTS equipped with an ultrashort NIR pulsed laser, a Michelson interferometer, a nonlinear crystal for MIR pulse generation and photodetectors. The NIR pulsed laser (Ti:Sapphire Kerr-lens mode-locked laser) generates ultrashort pulses centered at 800 nm with a pulse duration of 10 fs at a repetition rate of 75 MHz (Synergy Pro, Spectra-Physics). The NIR pulses are coupled into the Michelson interferometer after passing through a half-wave plate to generate double NIR pulses with an OPD between the first and second pulses, which is scanned by a motorized stage in the interferometer. The OPD is measured at interferometric precision by continuous-wave (CW) interferograms of a HeNe laser that monitor the motion of the scan mirror. The measured OPD is used for phase error correction of the interferograms. By using a polarization beamsplitter and quarter-wave plates to construct the Michelson interferometer, the double pulses generated from the Michelson interferometer are orthogonally polarized to each other. A long-pass filter at a cutoff wavelength of 700 nm (FELH0700, Thorlabs) and a chirped mirror pair (DCMP175, Thorlabs) tailors the NIR pulses spectrally and temporally. After the long-pass filter the spectral range of the NIR pulses spans from 10,870 to 14,340 cm^−1^.

### MIR pulse generation

The NIR double pulses are focused onto the 30-µm GaSe crystal (EKSMA OPTICS) using an off-axis parabolic mirror (OAPM) with a focal length of 25.4 mm for generating MIR pulses. The polarization of the focused pulses is adjusted with a half-wave plate. The pulse energy of the NIR pulse is 2–5 nJ, and that of the generated MIR pulse is tens of fJ. The remaining NIR pulses and generated MIR pulses are collimated by another OAPM with a focal length of 25.4 mm. Since the NIR pulses are slightly chirped by passing through the GaSe crystal, it is compensated by another chirped mirror pairs by separating the NIR and MIR pulses with a NIR/MIR dichroic mirror. The NIR and MIR pulses are combined again by another dichroic mirror. Here, to avoid spurious nonlinear effects at the sample, we intentionally have the NIR and MIR pulses separated in time. A pulse duration of the NIR pulses at the sample position is 11.9 fs evaluated by fringe-resolved autocorrelation measurement.

### Irradiation onto the sample

The NIR and MIR pulses are focused onto the sample by an OAPM with a focal length of 15 mm. The NIR pulse energies irradiated onto the sample are 1.1 nJ and 2.4 nJ for the first and second pulses, respectively. The liquid samples are contained in a cuvette made of 3-mm thick KBr windows, which is transparent in a wide spectral range covering both NIR and MIR. A teflon spacer with a thickness of 50 µm is inserted between the KBr windows for adjusting the sample thickness.

### Acquisition of the interferograms

The focused light onto the sample is collected and collimated by another OAPM with a focal length of 15 mm. After the collimation, the NIR and MIR pulses are spatially separated by a NIR/MIR dichroic mirror. The MIR pulses are detected by a N_2_-cooled MCT detector (KLD-0.5-J1-3/11, Kolmar Technologies) after passing through a linear polarizer, while the optically filtered NIR pulses are detected by an APD (APD410A2/M, Thorlabs), respectively. To detect the anti-Stokes scattering only, two short-pass filters (FESH0700, Thorlabs) are inserted in front of the APD. The detectors’ signals are low-pass-filtered and A/D-converted by a digitizer (ATS9440, AlazarTech). The CW interferogram of the HeNe laser is also digitized simultaneously. A part of the system where the MIR pulses travel through is enclosed by a box and purged with N_2_ gas in order to suppress undesired absorptions of the ambient gases, especially H_2_O vapor.

### Data processing

The sequentially measured interferograms are segmented into single interferograms and coherently averaged. The reference CW interferogram of HeNe laser is used for resampling the digitized interferograms. The MIR interferogram is processed as double-sided, while the CARS interferogram as single-sided. The MIR interferogram is retrieved numerically (See Supplementary Note 4 and Supplementary Fig. 3). A strong peak that appears at ZPD in the CARS interferogram caused by the non-resonant background is omitted from the Fourier-transform window. Both the interferograms are apodized by triangular function and Fourier-transformed. To show the IR transmittance, we measure spectra with and without the sample.

## Supplementary information

Supplementary Information

## Data Availability

The Matlab code used for analyzing the data of this study are available from the corresponding author upon reasonable request.

## Data availability

The data that support the findings of this study are available from the corresponding author upon reasonable request.
