# Supplementary material for: Complementary vibrational spectroscopy
Source: Nat Commun. 2019 Sep 27;10:4411. doi: 10.1038/s41467-019-12442-9 (PMC6764968; doi:10.1038/s41467-019-12442-9)
Supplement: Supplementary file 1 — Supplementary Information [file 41467_2019_12442_MOESM1_ESM.pdf]

Supplementary Information: Complementary Vibrational Spectroscopy

Kazuki Hashimoto[1,2], Venkata Ramaiah Badarla[3], Akira Kawai[1] and Takuro Ideguchi[*3,4]

[1] Department of Physics, The University of Tokyo, Tokyo 113-0033, Japan

[2] Aeronautical Technology Directorate, Japan Aerospace Exploration Agency, Tokyo 181-0015, Japan

[3] Institute for Photon Science and Technology, The University of Tokyo, Tokyo 113-0033, Japan

[4] PRESTO, Japan Science and Technology Agency, Saitama 332-0012, Japan

*ideguchi@gono.phys.s.u-tokyo.ac.jp

Supplementary Note 1: Detailed schematic of CVS

The full schematic of complementary vibrational spectroscopy (CVS) is depicted in Supplementary Fig. 1. The details of the system are described in Methods section.

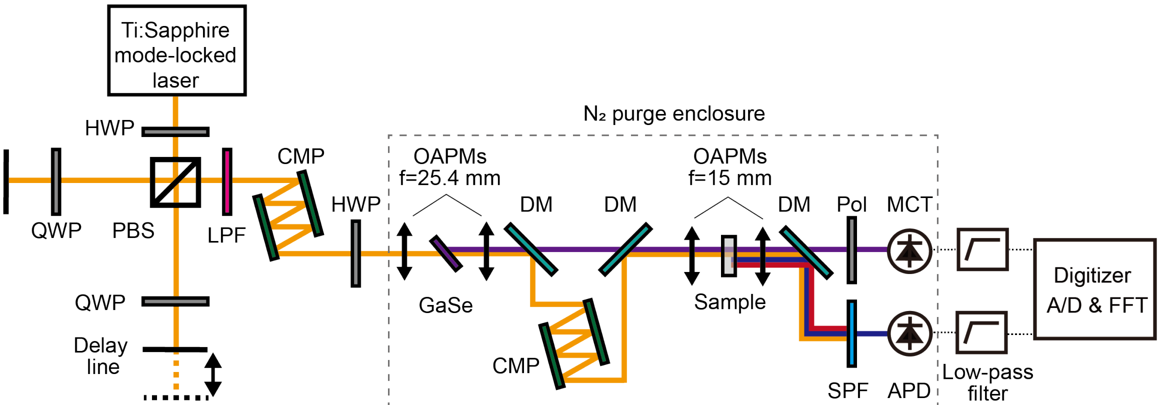

Supplementary Figure 1| Detailed schematic of CVS. HWP: Half-wave plate, PBS: Polarizing beamsplitter, QWP: Quarter-wave plate, LPF: Long-pass filter, CMP: Chirped-mirror pair, OAPMs: Off-axis parabolic mirrors, DM: Dichroic mirror, Pol: Polarizer, SPF: Short-pass filter, MCT: HgCdTe, APD: Avalanche photodetector.

Supplementary Note 2: Spectroscopic line assignment of the CVS spectra

We assign some major lines of the measured CVS spectra shown in Figure 4. There are vibrational modes in the CVS-Raman spectrum of benzene at 992 cm^{-1} (ring stretching), 3,063 cm^{-1} (C-H stretching)[1], and 2,950 cm^{-1} (combination or overtone band)[2], whereas in the CVS-IR spectrum at 1,036 cm^{-1} (C-H bending), 1,480 cm^{-1} (ring stretching+deformation)[1]. For chloroform, Raman lines at 667 cm^{-1} (C-Cl stretching) and 3,019 cm^{-1} (C-H stretching), and IR lines at 1,224 cm^{-1} (C-H bending) are observed[1]. For the 4:1 mixed sample of benzene and DMSO, the peaks derived from DMSO are observed at 666 cm^{-1} (C-S stretching), 2,915 cm^{-1} (C-H stretching) in the Raman spectrum[3], and at 947 and 1,054 cm^{-1} (S-O stretching + CH_3 rocking), at 1,307 cm^{-1} (umbrella) and 1,403 and 1,436 cm^{-1} (C-H

bending) in the IR spectrum[4] in addition to the specific peaks of benzene. Since there are some instrumental noise spikes in the CVS-Raman spectra, we evaluate them by comparing CVS-Raman spectra with and without the sample (Supplementary Fig. 2).

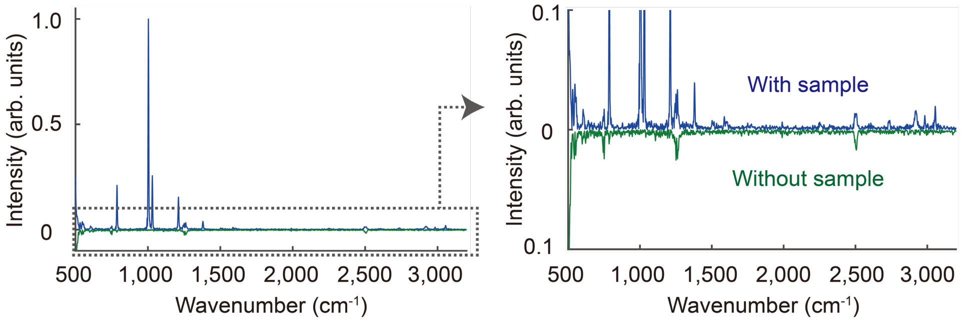

Supplementary Figure 2| Noise spike assignment in CVS-Raman spectrum. We evaluate the instrumental noise by comparing the obtained spectra with and without sample. The spectrum without sample is normalized with the noise peak at 500 cm^{-1}. The common peaks around 500 - 550 cm^{-1}, 750 cm^{-1}, 1,250 cm^{-1} and 2,500 cm^{-1} are attributed to the instrumental noise.

Supplementary Note 3: Comparison of multi-modal vibrational spectroscopy

Supplementary Table 1 shows comparison of the spectroscopic specifications of our CVS against the previously demonstrated multi-modal vibrational spectroscopy[5,6]. As clearly seen in the table, our CVS has broader spectral bandwidth spanning over 1,000 cm^{-1} in the fingerprint region with higher spectral resolution of around a few cm^{-1}, leading to the larger number of spectral elements than those of the previously demonstrated techniques.

There are advantages which make Fourier-transform spectroscopy (FTS) suitable for realizing the concept of CVS. First, FTS is known for a molecular spectroscopy technique with broader spectral coverage and higher spectral resolution compared with the other techniques such as grating-based dispersive or frequency-swept spectrometers. This capability originates from the noble interferometric measurement with a single detector. In reality, high-resolution and ultra-broadband molecular spectroscopy have been demonstrated with FTS-based systems both for infrared absorption and coherent Raman scattering spectroscopy[7, 8]. Second, FTS can gain a speed advantage. Especially, state-of-the-art FTS techniques such as ultra-rapid-scan FTS and dual-comb spectroscopy (DCS) drastically improve the spectral acquisition rate to for example 10 kHz without losing broadband and high-resolution capability. Third, FTS can provide more accurate frequency axis than the other methods due to the interferometric calibration with a reference continuous-wave laser. The accuracy can be ultimately up to the level of frequency standards when using a fully referenced dual-comb system. In the context of simultaneous measurement of IR absorption and Raman scattering spectra (complementary vibrational spectra), the frequency axes of the infrared and Raman spectra are calibrated at the same accuracy by a common interferometer, providing a big advantage for precise line position comparison of IR/Raman vibrational modes.

Supplementary Table 1 | Spectroscopic specifications of CVS and other multi-modal vibrational spectroscopy techniques. Spectroscopic specifications of CVS are compared with those of the other multi-modal vibrational spectroscopy in terms of spectroscopy method, observable spectral region, spectral bandwidth, spectral resolution and number of spectral elements.

Methods	IR spectroscopy / Raman spectroscopy	Observable spectral region (cm^{-1})	Spectral bandwidth (cm^{-1})	Spectral resolution (cm^{-1})	# of spectral elements
CVS (This work)	FT-IR	800-1,800	1,000	2 (Unapodized)	500
	FT-CARS	600-3,100*1	2,500*1	3.1 (Unapodized)	800
[Ref.5]	TSFG	2,750-3,000	~250*2	~20*3	~13
	CARS	2,750-3,000	~250*2	~20*3	~13
[Ref.6]	Spectral focusing IR	1,300-3,500*4	~2,200*4	~25*5	~88
	Spectral focusing CARS	2,250-3,250*4	~1,000*4	~20*5	~50

*1: The lowest and highest wavenumber of the observable spectral region of CVS is evaluated by those of the vibrational peaks obtained in this study. The lowest wavenumber is theoretically estimated to be ~100 cm^{-1}, leading to the theoretical spectral bandwidth of ~3,000 cm^{-1}.

*2: The spectral bandwidth may be expanded to ~2,400 cm^{-1} because the idler and signal tuning range of OPO (the laser source in Ref. 5) are 2,100-4,500 cm^{-1} and 5,000-7,400 cm^{-1}, respectively.

*3: The spectral resolution, limited by the linewidth of the picosecond lasers, is estimated to be ~20 cm^{-1} from the figure shown in Ref.5.

*4: The spectral range (and the bandwidth) of IR and Raman spectra is estimated from the figure shown in Ref. 6.

*5: The spectral resolution of ~25 cm^{-1} for IR and ~20 cm^{-1} for Raman spectra is estimated from the figure shown in Ref. 6.

Supplementary Note 4: Retrieval of MIR interferogram

The raw temporal mid-IR (MIR) waveform obtained by CVS contains several components including the linear MIR interferogram. The undesired components come from the additional MIR optical fields generated when the two near-IR (NIR) pulses are temporally overlapped in the nonlinear crystal. The situation is similar to the difference-frequency generation (DFG)-type interferometric (fringe-resolved) autocorrelation[9, 10]. Supplementary Fig. 3**a** shows the AC part of the waveform obtained by a CVS measurement. It consists of several components such as the intensity autocorrelation, high-frequency fringes in addition to the MIR interferogram[9]. Therefore, the Fourier-transformed spectrum shows the corresponding parts as shown in Supplementary Fig. 3**c**. The lowest frequency part of the spectrum near zero frequency appears due to the slowly-varying intensity autocorrelation, while the spectral components which appear around 12,500 cm^{-1} and 25,000 cm^{-1} come from the high frequency fringes corresponding

to the fundamental and second harmonic of the NIR pulses. The MIR spectrum appears at the spectral range of around 790 – 1,800 cm^{-1}. Since the MIR spectrum is well separated from the other spectral components in the frequency domain, we can clearly observe the MIR spectrum without the need for correction in our demonstration.

We can extract and retrieve the MIR interferogram by eliminating the unwanted components if necessary. Since the high-frequency spectra are well-separated from the MIR spectrum, low-pass filtering removes the high-frequency fringes. The intensity autocorrelation component can be removed by subtracting a fitted intensity autocorrelation curve (derived by $sech^2$ pulses) from the raw waveform. The retrieved MIR interferogram and its spectrum are shown in Supplementary Fig. 3**b** and 3**c**, respectively. This retrieval would be necessary especially when measuring lower frequency part, for example, less than ~850 cm^{-1} in our demonstration, where the MIR spectrum may overlap the low frequency spurious spectral component.

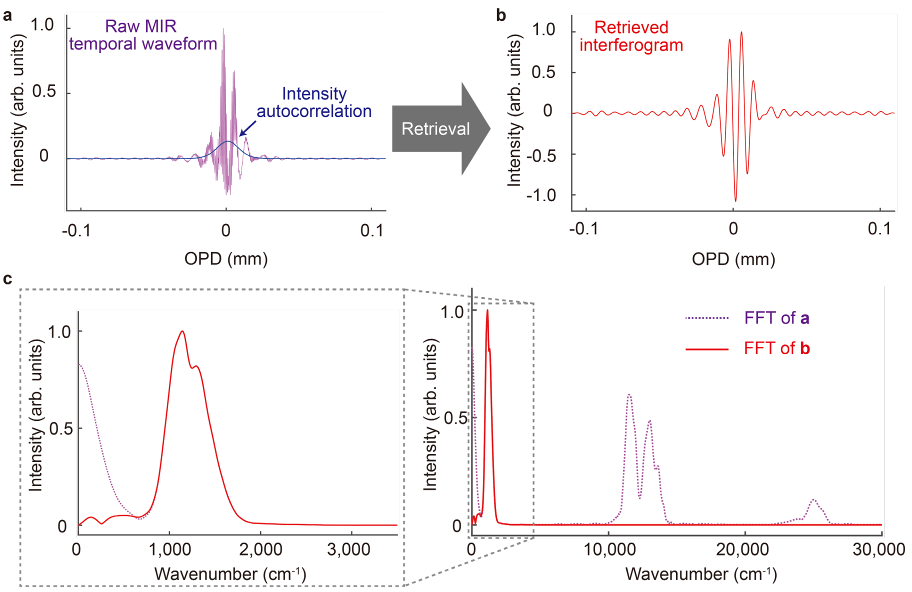

Supplementary Figure 3| Retrieval of MIR interferogram. a, AC part of the raw temporal waveform obtained by CVS. It consists of several components, which can be derived by DFG-type interferometric autocorrelation. OPD: optical path length difference. **b,** Retrieved MIR interferogram. **c**, FFT spectra of the raw temporal waveform (dotted curve in purple) and retrieved MIR interferogram (solid curve in red).

Supplementary Note 5: Sensitivity evaluation of CVS

We evaluate signal-to-noise ratio (SNR) of the CVS spectra of toluene. The SNR of the CVS-Raman signal (evaluated with the line at 1,003 cm^{-1}) increases quadratically to the total NIR pulse energy (the ratio of 1st and 2nd pulse energy is kept constant at 1:2) and reaches 380 at the pulse energy of 2.8 nJ and the measurement time of 0.2 s (Supplementary Fig. 4**b**). Here, the SNR is evaluated as ratio of a peak intensity of the Raman signal and a standard deviation where no peaks exist. We verify that the dominant noise is the relative intensity noise of the NIR pulses. The quadratic dependence on the excitation pulse energy can be explained by the cubic dependence of the signal intensity due to the $\chi^{(3)}$ nonlinear process (Supplementary Fig. 4**a**) and the linear dependence of the relative intensity

noise. On the other hand, SNR of the CVS-IR linearly increases to the average power of the MIR pulse and reaches above 150 at the average power of ~ 0.4 μW (estimated from the signal voltage of the detector and its responsivity) and the measurement time of 0.55 s (Supplementary Fig. 4**c**). The SNR of the CVS-IR spectrum is evaluated by the inverse of a standard deviation of the transmittance spectrum where no peaks exist. The detector noise is dominant in the condition of our demonstration. Note that the SNR of CVS-Raman/IR can be improved by optimizing the system and/or implementing some techniques such as heterodyne detection for CARS measurement.

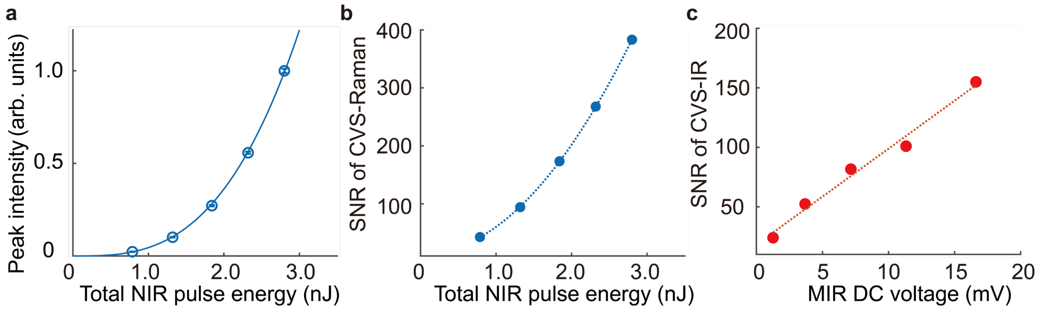

Supplementary Figure 4| SNR of CVS. a, Peak intensity of CVS-Raman signal at 1,003 cm^{-1} against total NIR pulse energy (solid blue line: fitting curve with a cubic function). Error-bars indicate standard deviation of the peak intensity. **b,** SNR of CVS-Raman signal at 1,003 cm^{-1} against total NIR pulse energy (dotted blue line: fitting curve with a quadratic function). **c**, SNR of CVS-IR spectrum against MIR DC voltage measured by a detector and an oscilloscope (dotted red line: fitting curve with a linear function).

Next, we evaluate detection limit of the CVS by measuring mixture of liquid benzene and chloroform. As shown in Supplementary Fig. 5**a**, the peak intensity of the CVS-Raman signal at 992 cm^{-1} shows linear dependence on the concentration, while the CARS process, in principle, is expected to show quadratic dependence. It is known that the heterodyne detection with a local oscillator can lead to linear dependence and we assume our FT-CARS is operated in a condition of the heterodyne detection with leaked NIR pulses through the optical filters. Estimating from the peak intensity and the noise level, the detection limit of benzene is to be ~0.1% with the measurement time of 0.2 s. Similarly, we measured absorbance of the CVS-IR spectrum with an isolated line of benzene at 1,394 cm^{-1} and confirm linear dependence on the concentration (Supplementary Fig. 5**b**). The detectable absorbance of this relatively weak absorption line is estimated to be ~4×10^{-3}, which corresponds to the density detection limit of ~2%, with the measurement time of 0.55 s. The detection limit is to be ~ 0.2% when observing the stronger absorption line at 1,480 cm^{-1}.

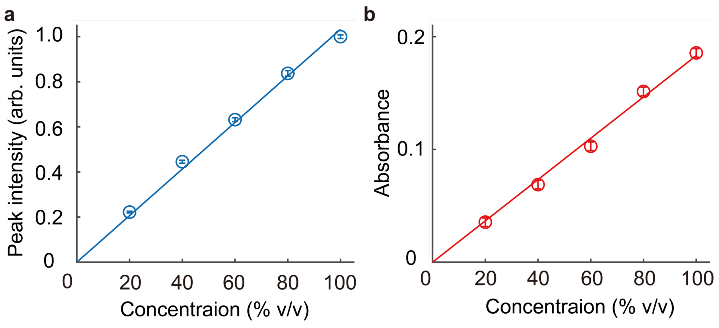

Supplementary Figure 5| SNR of CVS against concentration. a, Peak intensity of CVS-Raman signal at 992 cm^{-1} against the

concentration of benzene (solid blue line: linear fitting). Error-bars indicate standard deviation of the peak intensity. **b**, IR absorbance at 1,394 cm^{-1} against the concentration of benzene (solid red line: linear fitting). Error-bars indicate standard deviation of the absorbance where no absorption line exists.

Supplementary References

1. Shimanouchi, T. *Tables of molecular vibrational frequencies, consolidated volume I* (National Bureau of Standards, Washington, DC, 1972).
2. Hommei, E. L. & Allen, H. C. The air–liquid interface of benzene, toluene, m-xylene, and mesitylene: a sum frequency, Raman, and infrared spectroscopic study. *Analyst* **128**, 750-755 (2003).
3. Martens, W. N., Frost, R. L. Kristof, J. & Kloprogge, T. Raman spectroscopy of dimethyl sulphoxide and deuterated dimethyl sulphoxide at 298 and 77 K. *Journal of Raman spectroscopy* **33**, 84-91 (2002).
4. Skripkin, M. Y. et al. Vibrational spectroscopic force field studies of dimethyl sulfoxide and hexakis(dimethyl sulfoxide)scandium(III) iodide, and crystal and solution structure of the hexakis(dimethyl sulfoxide)scandium(III) ion. *Dalton Transactions* 4038-4049 (2004).
5. Hanninen, A. M., Prince, R. C. & Potma, E. O. Triple modal coherent nonlinear imaging with vibrational contrast. *IEEE J. Sel. Top. Quantum Electron.* **25**, 1-11 (2019).
6. Müller, N., Brückner, L. & Motzkus, M. Coherent Raman and mid-IR microscopy using shaped pulses in a single-beam setup. *APL Photonics* **3**, 092406 (2018).
7. Cossel, K. C. et al. Gas-phase broadband spectroscopy using active sources: progress, status, and applications. *JOSA B* **34**, 104-129 (2017).
8. Polli, D., Kumar, V., Valensise, C. M., Marangoni, M. & Cerullo, G. Broadband Coherent Raman Scattering Microscopy. *Laser Photon. Rev.* **12**, 1800020 (2018).
9. Joffre, M., Bonvalet, A., Migus, A. & Martin, J.-L. Femtosecond diffracting Fourier-transform infrared interferometer. *Optics Letters* **21**, 964-966 (1996).
10. Trebino, R., *Frequency-Resolved Optical Gating: The Measurement of Ultrashort Pulses* (Kluwer Academic, Dordrecht, 2000).
